# Solid-State Molecular Protonics Devices of Solid-Supported Biological Membranes Reveal the Mechanism of Long-Range Lateral Proton Transport

**DOI:** 10.1021/acsnano.3c11990

**Published:** 2024-02-05

**Authors:** Ambili Ramanthrikkovil Variyam, Mikhail Stolov, Jiajun Feng, Nadav Amdursky

**Affiliations:** † Schulich Faculty of Chemistry, Technion − Israel Institute of Technology, Haifa 3200003, Israel; ‡ Wolfson Department of Chemical Engineering, Technion − Israel Institute of Technology, Haifa 3200003, Israel

**Keywords:** membranes, proton transport, lateral diffusion, supported lipid bilayers, proton conduction

## Abstract

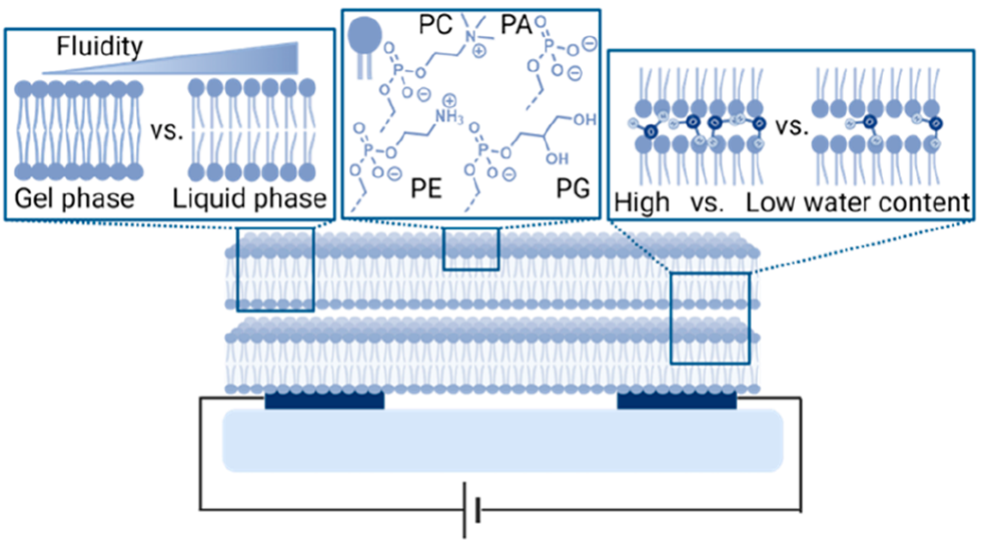

Lateral proton transport (PT) on the surface of biological membranes is a fundamental biochemical process in the bioenergetics of living cells, but a lack of available experimental techniques has resulted in a limited understanding of its mechanism. Here, we present a molecular protonics experimental approach to investigate lateral PT across membranes by measuring long-range (70 μm) lateral proton conduction via a few layers of lipid bilayers in a solid-state-like environment, i.e., without having bulk water surrounding the membrane. This configuration enables us to focus on lateral proton conduction across the surface of the membrane while decoupling it from bulk water. Hence, by controlling the relative humidity of the environment, we can directly explore the role of water in the lateral PT process. We show that proton conduction is dependent on the number of water molecules and their structure and on membrane composition, where we explore the role of the headgroup, the tail saturation, the membrane phase, and membrane fluidity. The measured PT as a function of temperature shows an inverse temperature dependency, which we explain by the desorption and adsorption of water molecules into the solid membrane platform. We explain our findings by discussing the role of percolating hydrogen bonding within the membrane structure in a Grotthuss-like mechanism.

## Introduction

Proton transport (PT) circuits involving biological membranes are of prime importance and are at the heart of our aerobic respiration system and of plant photosystems. When we discuss PT in the context of biological membranes, we need to differentiate between proton translocation across the two sides of the membrane, which is assisted by transmembrane proteins, and lateral proton diffusion on the surface of the membrane. Such lateral PT along membranes and its interface with the bulk aqueous environment were identified and distinguished from PT in water more than 25 years ago,^1−7^ and an apparent barrier exists across the bulk water–membrane interface, which affects proton activity at this interface.^3,8−22^ Several experimental works have shown the capability to observe lateral PT on the surface of biological membranes and that the membrane composition can affect this lateral PT.^10,23−28^ However, to date, most studies concerning long-range lateral PT (diffusion) on the surface of membranes, differentiated from short-range PT across the 5 nm bilayer, were of a spectroscopic nature and consisted of a molecular proton donor/acceptor situated at a certain position in the system. Unlike previous studies of membrane lateral PT, here, we present an in-depth exploration of the ability of membranes to support long-range lateral PT for distances of 70 μm using *electrical measurements*, i.e., following proton conduction in a solid-state type of molecular protonics device. As will be described below, the motivation and the added value for using electrical measurements are the capability to investigate the relation between PT to the number and structure of water molecules on the surface of the membrane.

The electrical properties of membranes have been investigated for many decades since the late 1960s, following the discovery of the model system that mimics the plasma membrane called black lipid membranes, for which the degree of ion impermeability across the lipid bilayers was evaluated in terms of ohmic resistance (on the order of a few MΩ·cm).^29−31^ During the 1980s, an additional membrane model was presented, which is the supported lipid bilayer (SLB).^32^ Due to their planar structure and compatibility with surface-based analytical methods, SLBs have become a platform for understanding the structure and physical characteristics of cell membranes^33−35^ and in biosensor applications.^36−38^ SLBs have also been characterized by electrical measurements across them, many utilizing impedance spectroscopy, particularly in understanding the interaction between membrane surfaces and their surroundings.^39−42^ In this context, we should also mention the widely used patch-clamp technique to follow the electrical resistance of biological membranes.^43−45^ Importantly, regardless of the chosen membrane model, the electrical measurements targeted the electrical response across the two sides of the membrane, i.e., through its nanometer-scale thickness and not its lateral parameters, and were performed with bulk water either on one side of the membrane (with SLB) or on both sides.

In this work, we present a different methodology to probe long-range lateral PT on the surface of biological membranes. Our methodology is based on following lateral proton conduction in a solid-state-like environment using molecular protonics devices composed of nanometer-scale SLBs, i.e., a solid sample in an environment with a certain relative humidity (RH). The solid-state environment enables direct focus on the role of the membrane surface and its composition in supporting lateral proton conduction while decoupling it from surface to bulk (or vice versa) events of PT. Thus, we can estimate the number of water molecules and their structure on the surface of the membrane and explore the role of water molecules within the membrane structure in supporting this long-range lateral proton conduction. Our temperature-dependence measurements of proton conductance show an interesting inverse temperature dependency; i.e., the conductance decreases as a function of temperature. We ascribe this peculiar finding to the number of water molecules on the surface of the membrane, which is yet another indication of the role of water molecules in assisting the lateral PT across the membrane. We discuss the possible proton conduction mechanism under different membrane conditions, where we show the importance of several lipid parameters, including headgroup, tail, phase, and fluidity, in supporting proton conduction due to its ability to form a percolating hydrogen bond network. In addition to the insights gained here into long-range PT on the surface of biological membranes, our findings of the specific role of the membrane interface in supporting lateral long-range PT are important to any use of membranes in various applications, from biomedical applications to the use of proton-conductive membranes in energy-related applications.

## Results

### Making and Characterizing the Supported Lipid Bilayers in the Solid-State

**Figure 1:**
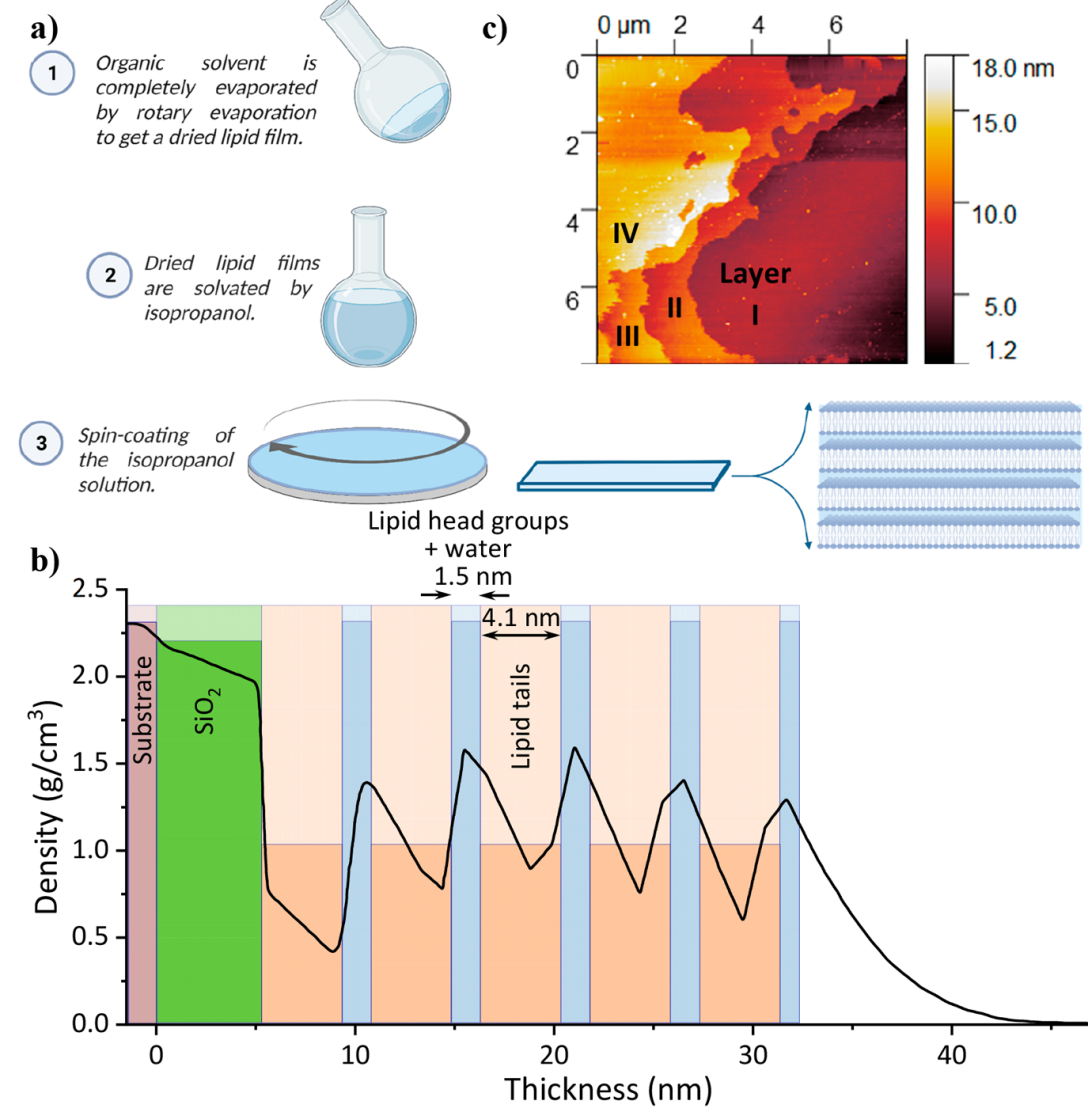
(a) General procedure of solid-state multi-SLB film preparation by spin coating of lipid solutions. (b) Density profile of a multi-SLB film as extracted from the XRR measurements (Figure S1). (c) AFM image of the edge of a multi-SLB film.

SLBs are generally prepared by using Langmuir–Blodgett film transfer or via vesicle fusion. These approaches produce a bilayer membrane on top of a hydrophilic surface such as Si oxide (SiO_*x*_) or glass in an aqueous environment.^46−48^ The presence of the aqueous environment is mandatory for the SLB configuration as it will collapse in a solid-state environment. As we aim here to probe lateral proton conduction in a molecular protonic solid-state device, we need to remove the aqueous phase from the system. To do so, we used spin-coating of a lipid solution on top of a SiO_*x*_ wafer, which resulted in a thin film of lipid bilayers (Figure 1a). To understand how many SLBs we have in our sample, we turned to various characterization techniques: ellipsometry, atomic force microscopy (AFM), and X-ray reflectivity (XRR). Both ellipsometry and XRR are reflection-based techniques used to characterize thin films. Ellipsometry measures changes in the visible light polarization upon interaction with a material, which is commonly translated to the thickness of the dielectric layer. In our ellipsometer measurements, we estimated the thickness of the spin-coated SLB film to be ∼20–25 nm, regardless of the lipid used to form the structure. The XRR is more sensitive to the internal ordering of the thin film. Our XRR measurements (Figure S1) clearly show a repeating structure within the thin film of 4–5 layers. The XRR measurements and fitting (Figure S1) can be used to extract the density profile of the thin film (Figure 1b), showing a repetitive pattern of low and high density for 4–5 layers, which we can ascribe to the lipid tail part and headgroup, respectively, whereas the latter also contains water molecules (*vide infra*). To complement these measurements, we turned to AFM, which can estimate both the thickness of the film and its uniformity. In Figure 1c, we show an AFM image of the edges of the spin-coated area that allows us to observe the individual layers within our multi-SLB. In line with our ellipsometer measurements, the AFM images also confirm that the thickness of the film is ∼20–25 nm. Thus, all indications point to a thin film of multi-SLB composed of 4–5 membrane bilayers, which represents the minimum number of layers for a stable multi-SLB film. It is very important to note in this stage that all lipids used in this study (Table S1) resulted in similar multi-SLB films with similar thicknesses. Nevertheless, it is also important to mention that due to the deposition technique of the multi-SLB using spin coating for a macroscopic surface area, we found some variance in the uniformity of the film in different areas of the macroscopic surface (Figure S2). As can be observed in Figure S2, whereas some areas are highly uniform, other areas contain patches within the surface, whereas all of the different lipids used in this study resulted in a similar pattern in the AFM measurements. The long-range proton conductance measurement averages the whole surface.

### Lateral Proton Conduction Across the Supported Lipid Bilayers in the Solid-State

**Figure 2:**
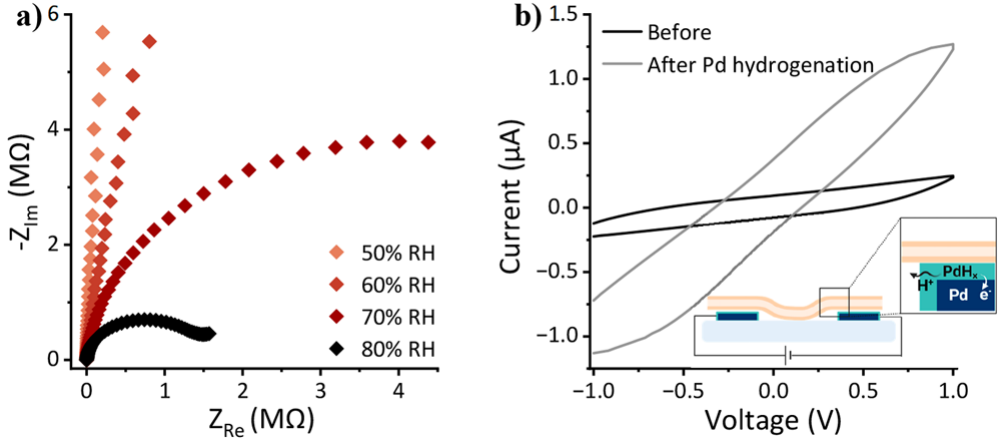
(a) EIS measurements of a device with a multi-SLB film at different relative humidities. (b) *I*–*V* measurements of a multi-SLB film before and after the formation of the hydrogenated Pd electrode. The inset shows a schematic of the PdH_*x*_ setup. Both panels show the results with DMPA SLBs (Table S1 for the chemical name and structure) measured at RT, whereas the RH-dependent EIS and *I*–*V* measurements of all other SLB films explored here are presented in Figure S5 and S6, respectively.

Following validating the fabrication of the multi-SLB films, we prepared them on an array of interdigitated metal electrodes made by Pd (for reasons that will be discussed below) on a SiO_*x*_ surface with a distance between electrodes of 70 μm. We measured the long-range conductance of the electrical device using both AC-bias-driven electrochemical impedance spectroscopy (EIS) and DC-bias-driven current–voltage (*I*–*V*) measurements. The importance of EIS measurements for estimating the bulk resistance properties of layers is due to their ability to decouple bulk charge resistance, which is the prominent process at high AC frequencies, from any processes occurring next to the surface of the electrodes that are prominent at low AC frequencies. Accordingly, to calculate the conductivity values of all lipids used in this study, we used the EIS results and fitted them to an equivalent circuit (Figure S4 and text within). It is worth mentioning that the conductivity extracted from a Pd-based device is similar to that extracted from multi-SLB films on the more common Au-based device (Figure S3 and discussed in ref (49)). It is important to note that an EIS or *I*–*V* measurement by itself is not enough to determine the nature of the charge carrier. However, we can already safely assume that proton conduction is the most likely source of the measured conductance, as any electronic conduction is very unlikely for such distances using an electronic-insulator-type material such as membranes, and any other ionic transport is also unlikely, as we did not introduce any ions to the system. Nonetheless, we present here two important experiments to determine the protonic conductance nature of our measurements. The first experiment is the RH-dependency of the EIS measurements (Figure 2a, shown as a Nyquist plot representation, imaginary part of impedance vs the real part, whereas the plots of all lipids are shown in Figure S5). In general, all “soft” proton conductors should exhibit a RH-dependency of their proton conductance, as water molecules are essential in supporting proton conduction through a percolating hydrogen bond network (further discussion below). As seen in Figure 2a, the impedance resistance is reduced as a function of RH, thus validating the role of protons as charge carriers across the membrane sample. The *I*–*V* measurements further show RH dependency (Figure S6) but with a smaller amplitude due to the discussed differences between these methodologies. The second important experiment to prove PT is performing *I*–*V* measurements with the Pd electrodes before and after their hydrogenation to PdH_*x*_ (Figure 2b). PdH_*x*_ electrodes are known for their capability to perform as proton transparent electrodes, meaning they can inject protons directly into the sample as opposed to the common electronic injection of metal electrodes.^50−53^ As shown in Figure 2b, the *I*–*V* measurements show a vast increase in the measured current following the formation of the PdH_*x*_ electrodes, which is an indication of a proton-conductive material. The hysteresis in the *I*–*V* measurements is due to the double-layer capacitive nature resulting from the presence of water molecules in our system.

### The Role of Membrane Parameters in Dictating Proton Conduction

**Figure 3:**
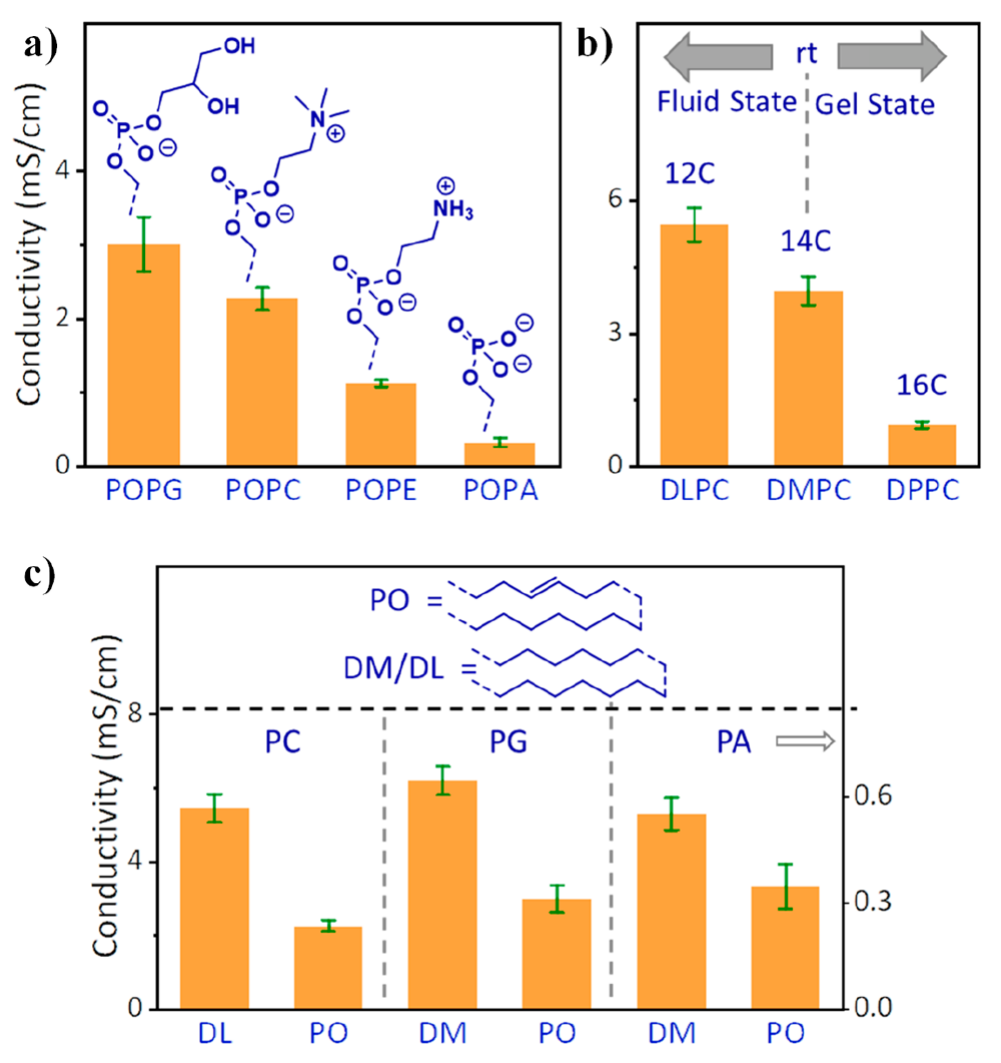
Conductivity at RT and 80% RH of all the different multi-SLB films under investigation as a function of (a) headgroup; (b) melting point/number of tail carbon atoms; and (c) tail saturation (the conductivity values of “PA” are on the right *y*-axis). The error bars represent the standard deviation of *N* > 3 samples for each studied membrane.

After confirming that protonic conduction is what we measure in our molecular protonic devices, we turned to decipher the role of different parameters associated with the membrane properties on the protonic conductivity of the multi-SLB film. Figure 3 summarizes the conductivity values of all lipids investigated here. The figure shows the measured protonic conductivity at 80% RH (at room temperature (RT), ∼23 °C), as it is the highest measured conductivity, but very importantly, the trends discussed below are identical for all of the different RH values (Table S2 for the conductivity values in different RH values probed). We can differentiate between the different lipids in terms of three important structural motifs:

1. Head groups (Figure 3a): In accordance with our hypothesis and in line with previous studies, the headgroup of a lipid should be a crucial component in the ability of the multi-SLB to support protonic conduction because of the capability of both the phosphate group and the charged/polar headgroup to participate in a hydrogen bond network. Here, we examine four distinct groups: phosphatidic acid (PA), phosphatidyl glycerol (PG), phosphatidylcholine (PC), and phosphatidylethanolamine (PE), where the first two are negatively charged and the latter two are zwitterionic (see schematic in Figure 3a and Table S1). Importantly, for a fair comparison, all the different lipids in this section shared the same unsaturated tail. From the impedance experiments and the extracted conductivity values, we can rank the conductivity values as follows: PG > PC > PE > PA (3.00 ± 0.36 > 2.26 ± 0.15 > 1.12 ± 0.04 > 0.32 ± 0.06 mS·cm^–1^, respectively). Our results point out the fundamental role of the headgroup in supporting long-range lateral proton conduction in a solid-state-like environment. Interestingly, the PA-terminated lipid is notably less proton conductive than the other bulkier lipids, highlighting the role of chemically “shielding” the phosphate group in the PT mechanism, which will be discussed below.
2. The melting point of lipids—Number of carbon atoms in the tail part (Figure 3b): For saturated lipids, the number of carbon atoms on the tail is directly related to the melting point. In this section, we compared the proton conductivity across membranes composed of lipids with PC head groups but differentiated by the number of saturated carbons in their tails: DLPC (12C), DMPC (14C), and DPPC (16C) with melting points of −2 °C, 23 °C, and 44 °C, respectively. As we measure at RT, DLPC is in its liquid state, DMPC is semiliquid, i.e., in the transition from liquid to gel, and DPPC is in its gel phase. Our results clearly show that a membrane in its liquid state is more conductive than a membrane in its gel phase. However, do all membranes in the same phase and with the same headgroup have a similar proton conductance? This question leads us to the next point.
3. Saturated vs unsaturated tail groups (Figure 3c): The level of saturation of lipid tails (i.e., whether the carbons are sp^3^ or sp^2^ hybridized) can have a vast effect on various membrane properties, primarily the fluidity of the membrane. For a fair comparison in this section, we compared two membranes with an identical melting point and a similar headgroup, where we chose POPC (unsaturated) and DLPC (saturated) having a melting point of −2 °C (Table S1); thus, they are liquid in RT. Our results indicate that the saturated membrane is more conductive than the unsaturated membrane. Importantly, our conclusion here that saturated membranes are more proton conductive than unsaturated membranes is also valid by comparing the saturated DMPA to the unsaturated POPA and the saturated DMPG to the unsaturated POPG. Although in these examples, the membranes do not share an identical melting point, they are in the same phase at RT. Our observation that membranes composed of saturated lipids are more conductive than membranes composed of unsaturated lipids is counterintuitive since membranes of unsaturated lipids are more fluid than membranes of saturated lipids, however as observed in the previous discussion, fluid liquid membranes are more conductive than gel membranes. Accordingly, this observation hints that there might be another parameter involved in proton conduction here, which leads us to the next discussion about the role of water molecules.

### The Role of Water in Proton Conduction

**Figure 4:**
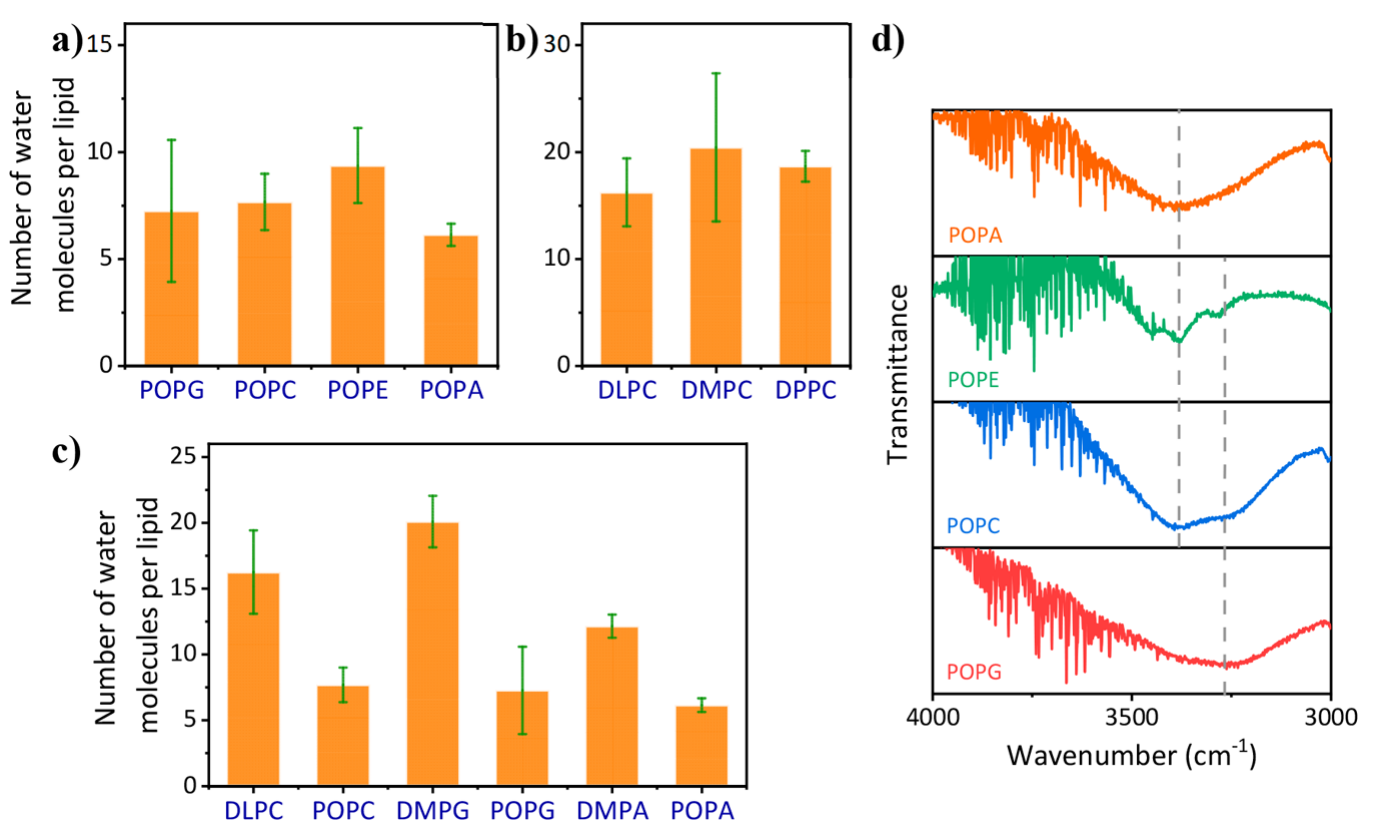
Results from the calculation of the number of water molecules per lipid at RT and 60% RH for all the lipid films as a function of (a) headgroup; (b) melting point/number of carbon atoms on tail and (c) tail saturation. (d) FTIR diagrams of lipid films as a function of their headgroup at RT and 60% RH showing vibrational bands between 3000 and 4000 cm^–1^. The error bars represent the standard deviation of *N* > 3 samples for each studied membrane.

In this section, we characterize the hydration of each multi-SLB, the amount of water molecules adsorbed to the multi-SLB structure following its formation, and the structure of the water molecule network within the membrane. Because of the solid-state nature of the multi-SLB structure, the amount of water molecules can be measured by weighing the final structure (see the Materials and Methods). Figure 4 shows the calculated number of water molecules per lipid molecule in each of the membranes measured in the conductivity measurements (as in Figure 3). When we compared the water content for membranes of lipids having the same unsaturated tail but different headgroups, we observed a similar water content (Figure 4a). Similarly, when we compared the water content for membranes of lipids with the same headgroup but different saturated tails, we also observed a similar water content (Figure 4b). These observations indicate that the change in proton conductivity measured in Figure 3a and 3b can be ascribed to the nature of the headgroup and the membrane phase, respectively. However, when we compared the water content for membranes of saturated vs unsaturated lipids having the same headgroup, we found a large change in water content, wherein membranes of saturated lipids had more water content than unsaturated lipids (Figure 4c). As will be discussed below, this important observation is the most likely explanation for the change in conductivity observed in Figure 3c.

Another important parameter of the water within the multi-SLB construct is related to the structure of the water molecules network within the membrane, which is an important factor in the ability of the material to transport protons in a Grotthuss-like mechanism (*vide infra*). To this end, we used FTIR, which is an important tool in discerning the hydration properties of lipid membranes.^54−60^ One of the prominent bands in the FTIR spectrum of hydrated lipid membranes is the classic O–H stretching band of water molecules centered at 3400 cm^–1^, which is sensitive to the extent of H-bonding in the film.^54^ Our FTIR measurements of the membranes in their multi-SLB configuration show that there are two main FTIR peaks in this region, and different membranes have different contributions from these two peaks (Figure 4d and Figure S7). These peaks are at 3400 and 3250 cm^–1^, whereas the former represents the classic O–H stretching band of water, while the latter is attributed to O–H stretching of water molecules in a large network of H-bonded structures.^54,57^ Accordingly, the ratio between these peaks is indicative of whether most of the water molecules are within a large network of H-bonded structures, thus exhibiting a predominant 3250 cm^–1^ peak, or they are not part of a network, thus exhibiting a predominant 3400 cm^–1^ peak. While comparing the FTIR spectra of the multi-SLB composed of POPG, POPC, POPE, and POPA, i.e., having the same tail at the same membrane phase but differentiated in their headgroup, we can see a different FTIR pattern (Figure 4d). Membranes of POPG exhibit only the 3250 cm^–1^ peak, thus indicating that the water molecules within them are mostly networked, whereas membranes of POPA exhibit only the 3400 cm^–1^ peak, thus indicating that the water molecules within them are mostly not networked. Membranes of the POPC and POPE show both peaks. Since we found that membranes of POPG are the most conductive and those of POPA are the least conductive (Figure 3a), the observation in this part points to the role of networked water in the ability of the membrane to be conductive. We further found that all PC-terminated membranes have the discussed two FTIR peaks, PA-terminated membranes have only the 3400 cm^–1^ peak and PG-terminated membranes have only the 3250 cm^–1^ peak (Figure S7), but it is more complicated to compare the FTIR measurements for membranes having different phases or fluidity.

### The Effect of Temperature on Proton Conduction

**Figure 5:**
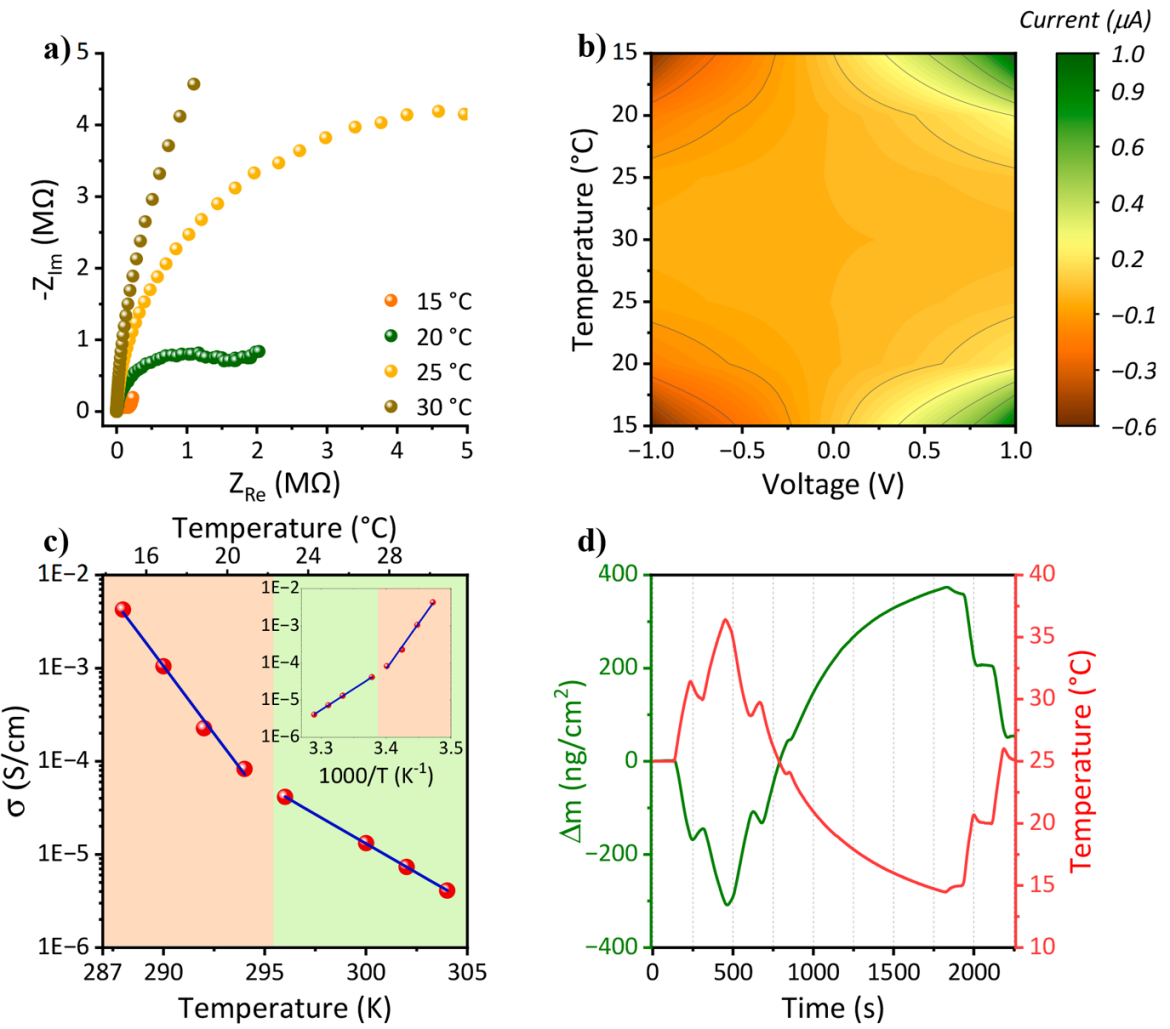
Temperature-dependent electrical measurements of multi-SLB films using (a) impedance measurements and (b) *I*–*V* measurements. The graph in panel (b) shows the reversibility of protonic currents on the heating and cooling cycle, while the current is displayed as a heatmap. Panels (a) and (b) show the results for the POPC multi-SLB film, whereas the EIS and *I*–*V* measurements for all lipids are displayed in Figure S8 and S9, respectively. (c) The extracted conductivity as a function of temperature for the DMPG multi-SLB film covering the range of the two membrane phases: gel and liquid, marked with orange and green rectangles, respectively. Similar graphs for all other multi-SLB films are presented in Figure S11. The inset shows the same on a 1000/*Tx*-axis. (d) QCM measurement of a DMPG multi-SLB film upon one cycle of heating and cooling (Figure S12 shows multiple cycles). The measuremts were done at 60% RH.

In general, proton conduction, regardless of the mechanism, should be a thermally activated process, meaning that the conductance should increase with temperature. To our surprise, we observed an inverse temperature dependency in both our impedance (Figure 5a and Figure S8 for all lipids) and *I*–*V* (Figure 5b and Figure S9 for all lipids) measurements, i.e., the measured conductance/current decreased as a function of temperature. The temperature range that we used is 15 to 35 °C to avoid condensation of water molecules that started to appear below the range, while above this temperature range, the conductivity reached a very small value.

Importantly, this inverse temperature dependence is reversible for several cycles of heating and cooling (Figure 5b and Figure S10). While plotting the dependency of the extracted proton conductivity from the EIS measurements as a function of temperature (Figure S11 for all of the lipids), we can see a trend for the decrease in extracted conductivity with temperature. Specifically, for DMPG and DMPC, we extended our temperature range to have the melting point temperature of these SLBs (∼23–24 °C) in the middle of the measured temperature range, thus allowing us to explore the role of the membrane phase in the temperature dependency of proton conduction. We can see that the different membrane phases, i.e., liquid vs gel phases, have different dependencies on temperature (Figure 5c, green and orange areas, respectively). Importantly, the dependency of conduction on temperature is approximately 2-fold larger for the gel phase than for the liquid phase, meaning that for the gel phase, a small temperature change results in a larger change in conductivity than in the liquid phase. It is also important to note that the changes in the conductivity for the different lipid characteristics, as presented in Figure 3, remain similar at different temperatures (Table S3). It is worth noting that while measuring at room temperature, membranes of gel phase have poorer proton conduction than those of liquid phase (as discussed in Figure 3b), and in the temperature-dependence study we observe that for a given membrane, the low-temperature gel phase is more conductive than the high-temperature liquid phase. The latter finding highlights that there must be an additional factor that determines the efficiency of proton conduction, and as will be discussed in the next section, it is the water molecules.

To rationalize the inverse temperature dependence of protonic conduction, we conducted several types of experiments. First, we used temperature-dependent XRR measurements to follow any noticeable changes in the thickness of the multi-SLB film or its internal structure. As shown in Figure S13, we observed no change in the XRR pattern at different temperatures, thus indicating that the observed inverse temperature dependence of the measured proton conduction is not due to a change in the membrane structure within the SLB configuration.

Since water molecules have an important role in the ability to support long-range proton conduction by assisting in forming hydrogen bond networks, the next immediate suspect to rationalize the inverse temperature dependence is the number of water molecules in the multi-SLB film. For that measurement, we used temperature-dependent quartz crystal microbalance (QCM) measurements to estimate the change in the mass of the films at different temperatures. We detected a reversible mass change of the film as a function of temperature, where increasing the temperature resulted in a decrease in mass (Figure 5d) and vice versa for numerous cycles (Figure S12). In our QCM measurements, we can safely conclude that the observed change in mass is due to the presence of water molecules. Thus, the role of water molecules in supporting the observed proton conduction in a mechanism that will be described below is highlighted.

### Does Proton Conduction Happen Across the Entire Cross-Section of the Multi-SLB Film or Not?

**Figure 6:**
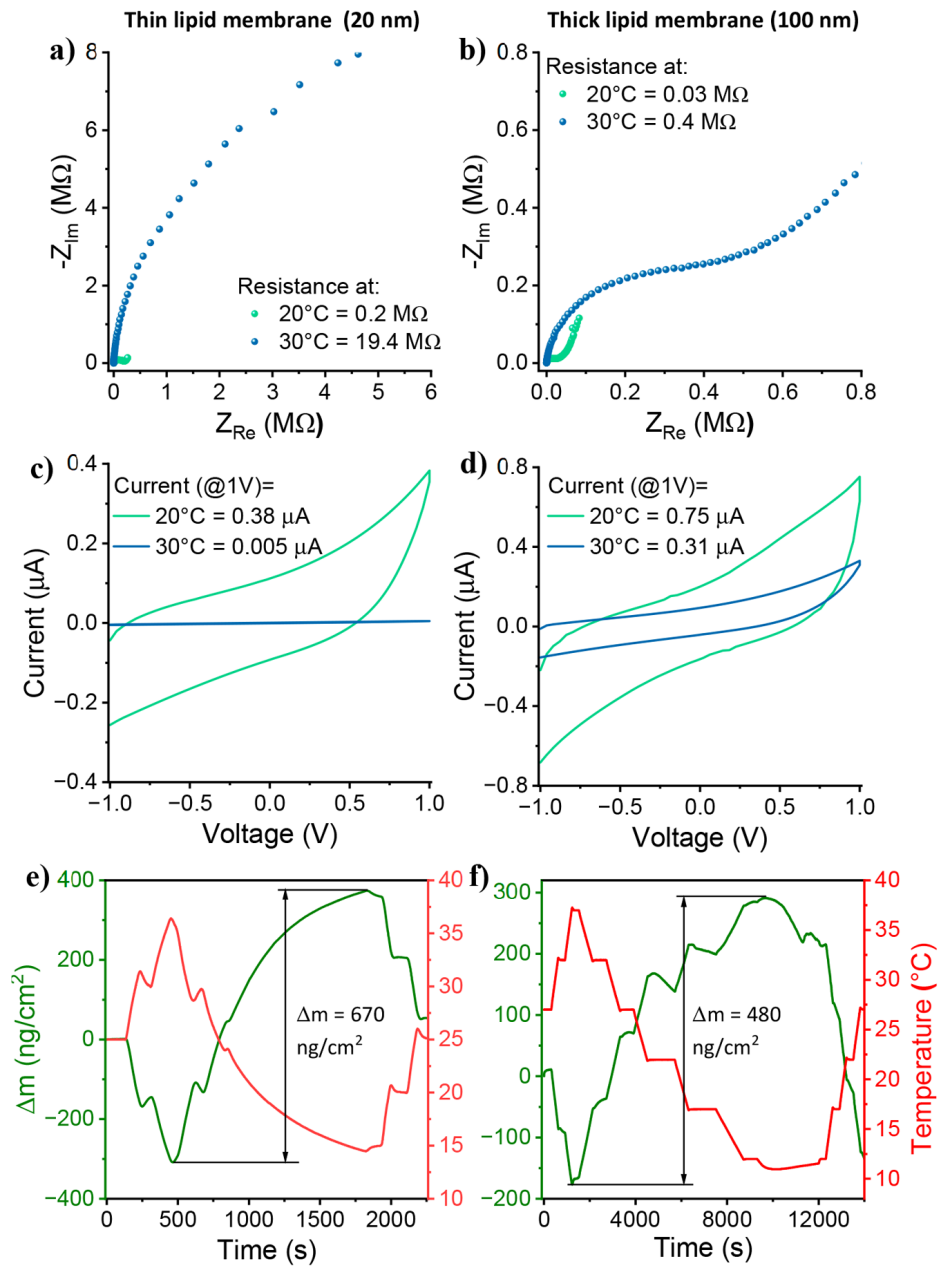
(a,b) Impedance measurements and (c,d) *I*–*V* measurements of a thin (20 nm) (a,c) and a thick (100 nm) multi-SLB film (b,d) at two different temperatures. (e,f) Temperature-dependence QCM measurements of the thin and thick multi-SLB films, respectively. All measurements here were conducted with DMPG SLBs. The measuremts were done at 60% RH.

To answer this question, in the last part of our proton conduction measurements, we investigated the role of film thickness and its temperature dependence on the measured proton conductivity. In accordance with our described methodology, the thickness of the multi-SLB film can be easily tuned by varying the rpm and time of the spin-coating process (the 20 nm multi-SLB film discussed thus far represents the minimum thickness). Thickness-dependent measurements can resolve if the conductance is occurring through the entire cross-section of the membrane or just next to the bottom surface, which is in contact with the electrodes at its edges. As shown in Figure S14, the measured conductance increases as a function of thickness, but the measured conductivity is similar regardless of the thickness. This implies that the measured protonic transport occurs through the entire cross-section of the film and that all of the bilayers in the multi-SLB structure contribute to the measured proton conductance. Nevertheless, the temperature dependency is different between different thicknesses, whereas the thicker the film is, the smaller the (inverse) temperature dependency, as observed both in the EIS measurements (Figure 6a,b) and in the *I*–*V* measurements (Figure 6c,d). To explain this result, we compared the temperature dependence QCM measurements between the thin and thick samples (Figure 6e,f), where we observed a relatively similar order of change in mass between the samples (even smaller changes in the thick sample). This indicates that the amount of water loss and gain per mass of lipids is much smaller in the thick sample than in the thin sample, which is translated to a much smaller change in the conductance as a function of temperature.

## Discussion

In the discussion section, we intend to detail our suggested PT mechanism across the multi-SLB films composed of different lipids that can explain our presented results. First, we need to discuss the conventional PT mechanism across soft biological materials in a solid-state-like environment. We define such an environment as having a solid sample that contains a certain amount of water, and the environment is at a certain RH. To date, most of the reported soft biological materials that show protonic conductivity in these solid-state conditions are either composed of proteins (or peptides) or polysaccharides.^50−53,61−70^ In terms of measured conductivity, most of the multi-SLB films used in this study are at the upper end of reported conductivity values for proteins and polysaccharides, which are typically in the range 0.01–1 mS·cm^–1^. The common long-range PT mechanism suggested for all solid-state biological materials is the Grotthuss mechanism. This mechanism is based on proton hopping along a hydrogen bond network. It was shown in several studies that in solid-state biological materials, this network is composed of both water molecules within the material and specific polar or charged moieties of the biological entity, which are the amino acid residues in proteins or the polar groups in glycan chains.^51,62,64,67−69,71,72^ Accordingly, the hydrogen bond network is facilitated within the interface of the biological material, and the trapped water molecules are bound to it. It is also important to note that this mechanism is similar to the common convention of hydrogen bond networks within natural proteins capable of proton translocation, such as the transmembrane proteins in photosynthesis or the aerobic respiration system. In addition to the Grotthuss mechanism, the main other suggested mechanism for soft matter in the solid-state is the vehicular mechanism, which is the movement of ions (such as proton hydrate) via a vehicle that is usually water molecules surrounding the ion. To facilitate this type of mechanism, there is a need for large water cavities within the material, which is not the case in multi-SLB film. Hence, in line with all other biological materials, the Grotthuss mechanism is the suggested mechanism in our case.

Now, to discuss the specific hydrogen bond network responsible for the measured proton conduction, we refer to our measurements with different lipid headgroups (Figure 3a). In these measurements, we observed that PT across PA-terminated SLBs is significantly poorer than the other lipids used. This is somewhat in contrast to measurements performed with single lipid bilayers in solution (in the form of a vesicle), where it was shown that PA lipids can facilitate PT via surface-to-bulk or bulk-to-surface interactions.^25,28^ In our case, we do not have a bulk solution; hence, there are no such interactions. In contrast, our observation that the additional chemical moieties, regardless of whether they were charged or not, on top of the phosphate group, resulted in an increase in PT compared to the bare PA points to a “shielding” effect. Accordingly, the only rational mechanism to explain it is the presence of a hydrogen bond network of trapped water molecules and the phosphate backbone of the lipids for the formation of shielded network wires. In that sense, a polar moiety (PG-terminated) is more efficient in the formation of the hydrogen bond network than a positively charged group (PC-terminated), and a bulkier group (PC) is more efficient than a less bulky group (PE-terminated, which is also positively charged). Our FTIR measurements (Figure 4d) highly support this explanation by showing that water molecules within PG-terminated membranes are in a network configuration, while in PA-terminated membranes, they are not. In this context, it is important to note earlier works that discussed the importance of water wires in the ability to facilitate PT in high efficiencies, either within proteins or even on the surface of the membrane.^73−78^ Also, the significance of the orientation of water molecules relative to the bilayer membrane and the interaction of proton hydrate with the headgroup of lipids have been well explored in the past.^5,6,10,79,80^

In terms of temperature dependency, the Grotthuss mechanism should be thermally activated with a commonly measured activation energy on the order of 0.1–0.2 eV,^73^ which is based on the basic collision theory and transition state theory, for which with increasing temperature, the rate of charge transfer will increase (negative Δ*G*^‡^). However, we observed an inverse temperature dependency (a positive slope in the Arrhenius/Eyring equation). To explain this, we should realize that our multi-SLB films contain a low amount of water, and as such, a small amount of water loss can have an enormous effect on the intactness of the hydrogen bond network, which will result in a poor PT across the material (as observed). In our case, heating induces the desorption of water molecules, while cooling results in their reabsorption from the humid environment. In other words, at different temperatures, the composition of the system changes in terms of the number of water molecules, thus explaining the deviation from the basic collision theory for charge diffusion. Our results of a less drastic inverse temperature dependency of the thicker multi-SLB films support this hypothesis. Also, the observation that, in general, the drop in conductance for membranes that contained more water molecules is larger as a function of temperatures than membranes that contained less water molecules (Table S3) further supports our conclusion.

The last point in the mechanism discussion is related to the effect of the membrane phase and membrane fluidity on the PT properties. Here, we made two important observations. (1) While measuring at a certain temperature (RT), PT across a gel phase of the membrane is less efficient than PT across its liquid phase (Figure 3b). Here, we can safely claim that there is a need for membrane flexibility, which is present only in the membrane liquid phase, to facilitate the formation of a percolating hydrogen bond network within the discussed shielded wire composed of phosphate backbones. Our observation of a different thermal response of measured conductivity at different phases of a certain membrane supports this notion (Figure 5c). (2) PT across a more fluid membrane composed of unsaturated lipids is less efficient than PT across a less fluid membrane composed of saturated lipids having the same headgroup (Figure 3c). This observation is counterintuitive, as we would expect that a more fluid membrane will have better PT capabilities than a less fluid membrane. On the other hand, we observed a different water content for these two membrane compositions, whereas saturated lipids contain more water in the multi-SLB configuration than unsaturated lipids. Accordingly, the changes in the PT efficiency here should be explained in terms of the amount of water molecules, i.e., more water molecules and a better network of hydrogen bonds.

Overall, all experimental indications point to a sweet point for the most efficient PT across solid membrane films: We want the membrane to be in its liquid state with a high water content and with a bulky polar headgroup. This is the reason the saturated DMPG membrane with a melting point below RT is the most conductive film in our study.

## Conclusions

In summary, we designed and created a model of a multi-SLB film composed of 4–5 bilayers of membranes in a solid-state-like environment to investigate lateral proton conduction over long distances across membranes in a molecular protonics device. Proton conduction was confirmed by both RH-dependent measurements and the use of proton transparent electrodes. In terms of how the composition of the membrane affects the measured proton conductivity, we found that (1) the lipid headgroup is of prime importance with the following trend in proton conductivity: PG > PC > PE > PA; (2) the lipid phase influences the measured conductivity, wherein fluid membranes conduct better than gel membranes; and (3) membranes of saturated lipids conduct better than unsaturated lipids even though membranes of unsaturated lipids are more fluid than saturated ones. By measuring the water content and the water structure within the multi-SLB configuration, we could rationalize why membranes of unsaturated lipids are poorer proton mediators compared to membranes of saturated lipids. We show that the most conductive PG-terminated membrane has water molecules in a network structure, while the least conductive PA-terminated membrane does not have such a network. In terms of how temperature affects the measured proton conductivity, we found an inverse temperature dependency for all investigated membranes. QCM measurements have resulted in our understanding that the desorption/adsorption of water molecules from the multi-SLB film is the main factor responsible for the observed inverse temperature dependency. In terms of the PT mechanism, we discussed how the Grotthuss mechanism can be applied to our results and how the change in the different parameters can influence the percolating hydrogen bond network across the membrane surface that is needed for an efficient PT.

## Materials and Methods

### Sample Preparation

Each lipid was dissolved in chloroform, followed by solvent evaporation using a rotary evaporator until a dried lipid film was formed. The lipid film was kept overnight inside the vacuum chamber and was redissolved in isopropanol to a lipid concentration of 3 mg/mL.

### Electrode Preparation

The devices were prepared by using silicon wafers with a SiO_*x*_ dielectric layer (110 nm). Au/Pd electrodes (200 nm) were deposited on 40 nm Cr on top of the substrates through a shadow mask using a thermal evaporator at a deposition rate of 2 Å s^–1^ under 5 × 10^–7^ Torr for the making of interdigitated electrodes (distance between electrodes of 70 μm).

### Spin Coating

100 μL portion of the lipid-containing isopropanol solution was added to a silicon wafer and spin-coated at a rate of 2000 rpm for 120 s using a spin coater (EZ – spin A1, Apex instruments). Following spin coating, the sample was dried by using a nitrogen stream.

### Electrochemical Impedance Spectroscopy

Proton conductivity measurements were carried out using an impedance/gain-phase analyzer (MTZ-35, Bio-Logic). Lipid samples were spin-coated onto the prepared electrodes. The electrodes were made to be in contact using a probe station micromanipulator. A 50 mV AC bias was applied during the measurements, and a frequency range of 10 MHz to 10 Hz was used for the experiments. Temperature-dependent studies were performed using a Peltier-containing probe station (INSTEC) in the range of 15 to 35 °C. The conductivity of the films was calculated using the following equation: *G* = σ*A*/*l*, where *G* is the conductance (as extracted using the equivalent circuit), σ is the conductivity, *A* is the cross-sectional area of the lipid film (*A* = thickness of film × width of the film), and *l* is the distance between two electrodes. For each condition, more than three different samples were measured to calculate the standard deviation of the results.

### Current–Voltage Measurements

Current–voltage measurements (*I*–*V*) were carried out using a source-measuring unit (B2912A, Agilent). Lipid films were placed on top of the Au/Pd electrodes for the *I*–*V* measurements. The current was measured as a function of voltage between −1 and 1 V at a scan rate of 100 mV s^–1^. Hydrogen gas was supplied into the probe station for approximately 30 min to create hydrogenated Pd electrodes. The temperature-dependent studies were conducted as mentioned above.

### Atomic Force Microscopy

AFM measurements (XE-100 AFM, Park Systems) were performed in tapping mode using NSG30 AFM probes (spring constant of ∼40 N/m) below the resonance frequency (typically, 320 kHz) under ambient conditions at room temperature. The resulting images were processed by using Gwyddion software.

### Ellipsometry

Ellipsometry experiments (ALPHA-SE ellipsometer, J.A. Woollam Co.) were carried out at an incident angle of 70° with respect to the surface normal.

### Fourier Transform Infrared (FTIR) Spectroscopy

FTIR measurements (Tensor 27 spectrometer, Bruker) were acquired in the range of 400 to 4000 cm^–1^. For each sample, the background was measured and subtracted from the spectrum. The samples were prepared using drop casting of a lipid solution on a silicone surface.

### Number of Water Molecules Per Lipid

Thick multi-SLB films were formed as described above on SiO_*x*_. The mass of each lipid film was measured using a microbalance to calculate the amount of water associated with each of the films at RT and 60% RH.

### X-ray Reflectometry

The XRR measurements were carried out using the X-ray reflectivity mode of a Rikagu SmartLab high-resolution diffraction system.

### Quartz Crystal Microbalance

Before the experiment, gold-plated QCM sensors (Renlux Crystal, 5.0 MHz resonant frequency) were prepared as described previously.^81^ Sensors were first cleaned in a UV-ozone chamber (ProCleaner, BioForce Nanosciences) for 15 min, sonicated in 98% ethanol for 15 min, rinsed in ultrapure water for 3 min, dried with nitrogen, and cleaned again in UV-ozone for 15 min. Frequency shifts Δ*f* were recorded for the 3rd, 5th, 7th, 9th, 11th, and 13th overtones using a Q-Sense E4 (Biolin Scientific) module. Δ*f* was recorded in the air for the blank and coated sensor to estimate the attached film mass using the Sauerbrey equation.^82^ The experiment was conducted in two humidity regimes: dry nitrogen or nitrogen saturated with water vapor. The flow of appropriate gas was purged through the series of two QCM cells with coated and reference sensors at a 100 μL/min rate using an IsmaTec peristaltic pump (IDEX) both during the measurement and for at least three h beforehand for equilibration. During the measurement, the temperature was varied according to the programmed loop. The change in the sample mass was calculated using the Sauerbrey equation.

## Supporting Information Available

Chemical names, structures, and melting points of all the lipids; Conductivities of all SLBs for different RHs at RT; Conductivities of all lipids for two temperatures and at 60%; XRR of DMPC membrane; AFM images of multi-SLBs on silicone; Nyquist plot from EIS measurements of DMPG on Au and Pd electrodes; Equivalent circuit used for fitting Nyquist plots; Impedance measurements of all lipids at different RHs; *I*–*V* diagram of all SLBs at different RHs; FTIR diagram of membranes of saturated lipids; Temperature dependent impedance measurements of all lipids; Temperature dependent *I*–*V* of all lipids; *I*–*T* plot showing the reversibility of currents produced by lipid films over heating and cooling cycles; Conductivity vs temperature plots for all lipids; QCM measurement showing reversible change in the mass of lipid films over six consecutive heating and cooling cycles; XRR diagram of DMPG membrane at different temperature; Nyquist plots of DLPC films of three different thickness (PDF)
