# Supplementary material for: Solid-State Molecular Protonics Devices of Solid-Supported Biological Membranes Reveal the Mechanism of Long-Range Lateral Proton Transport
Source: ACS Nano. 2024 Feb 5;18(6):5101–12. doi: 10.1021/acsnano.3c11990 (PMC10867892; doi:10.1021/acsnano.3c11990)
Supplement: Supplementary file 1 — nn3c11990_si_001.pdf [file nn3c11990_si_001.pdf]

Supporting Information

Solid-state molecular protonics devices of solid-supported biological membranes reveal the mechanism of long-range lateral proton transport

Ambili Ramanthrikkovil Variyam,[a] Mikhail Stolov,[b] Jiajun Feng,[a] and Nadav Amdursky[a,*]

[a]Schulich Faculty of Chemistry, Technion – Israel Institute of Technology, Haifa 3200003, Israel.

[b]Wolfson Department of Chemical Engineering, Technion – Israel Institute of Technology, Haifa 3200003, Israel.

*Corresponding author email: amdursky@technion.ac.il

Table S1: The table summarizes the chemical name, structure, and melting point of all the lipids investigated in the paper.

Lipid	Chemical Name	Melting Point (°C)	Structure
**DLPC**	1,2-dilauroyl-sn-glycero-3-phosphocholine	-2	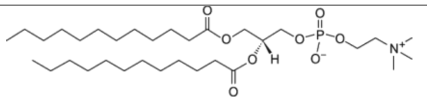
**DMPC**	1,2-dimyristoyl-sn-glycero-3-phosphocholine	24	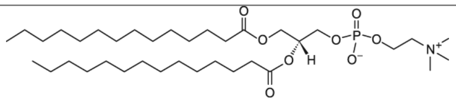
**DPPC**	1,2-dipalmitoyl-sn-glycero-3-phosphocholine	41	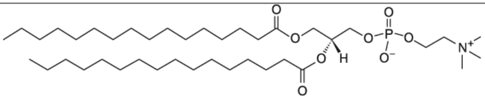
**DMPA**	1,2-dimyristoyl-sn-glycero-3-phosphate	52	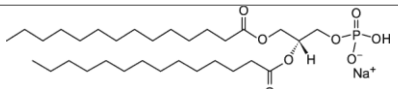
**POPC**	1-palmitoyl-2-oleoyl-glycero-3-phosphocholine	-2	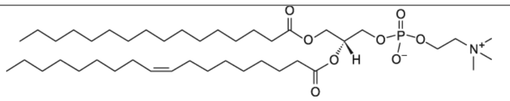
**POPA**	1-palmitoyl-2-oleoyl-sn-glycero-3-phosphate	28	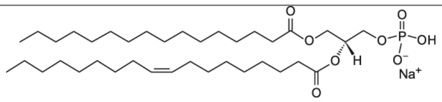
**POPG**	1-palmitoyl-2-oleoyl-sn-glycero-3-phospho-(1'-rac-glycerol)	-2	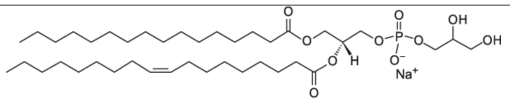
**DMPG**	1,2-dimyristoyl-sn-glycero-3-phospho-(1'-rac-glycerol)	23	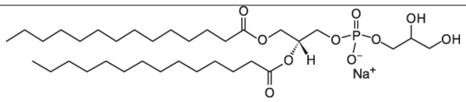
**POPE**	1-palmitoyl-2-oleoyl-sn-glycero-3-phosphoethanolamine	25	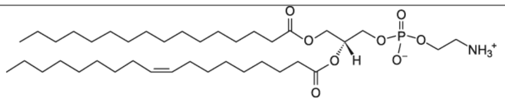

Table S2: Measured conductivities of all SLBs at different RH conditions. Measurements were carried out at RT. N>3 for the number of samples for each studied membrane.

**Lipid Membrane**	**80% RH**		**70% RH**		**60% RH**	
	**σ (mS/cm)**	**Error (±)**	**σ (mS/cm)**	**Error (±)**	**σ (mS/cm)**	**Error (±)**
DMPG	6.2	0.4	1.74	0.21	0.28	0.03
DLPC	5.4	0.4	0.91	0.18	0.22	0.01
DMPC	4.0	0.3	0.51	0.07	0.15	0.01
DPPC	0.93	0.07	0.10	0.01	0.037	0.003
DMPA	0.53	0.04	0.055	0.009	0.027	0.002
POPA	0.32	0.06	0.033	0.005	0.021	0.024
POPE	1.12	0.04	0.16	0.02	0.047	0.006
POPC	2.27	0.15	0.21	0.01	0.076	0.010
POPG	3.0	0.4	0.31	0.02	0.096	0.005

Table S3: Measured conductivities of all membranes at two different temperatures and at the same 60% RH condition. N>3 for the number of samples for each studied membrane.

**Lipid Membrane**	**15 °C**		**25 °C**	
	**σ (mS/cm)**	**Error (±)**	**σ (mS/cm)**	**Error (±)**
DMPG	16.4	2.1	0.24	0.03
DLPC	7.0	0.4	0.18	0.01
DMPC	5.4	0.7	0.11	0.01
DPPC	0.52	0.08	0.029	0.003
DMPA	0.30	0.02	0.020	0.001
POPA	0.21	0.02	0.016	0.001
POPE	0.98	0.32	0.045	0.005
POPC	1.49	0.09	0.059	0.007
POPG	2.73	0.19	0.092	0.008

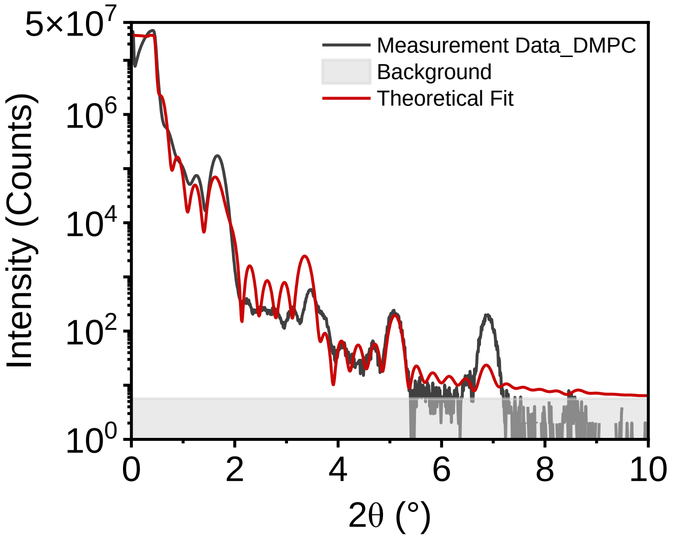

Figure S1: XRR result of DMPC at room temperature together with the best fitting of the XRR curve indicating 4-5 bilayers of lipid membrane on top of a silicon wafer.

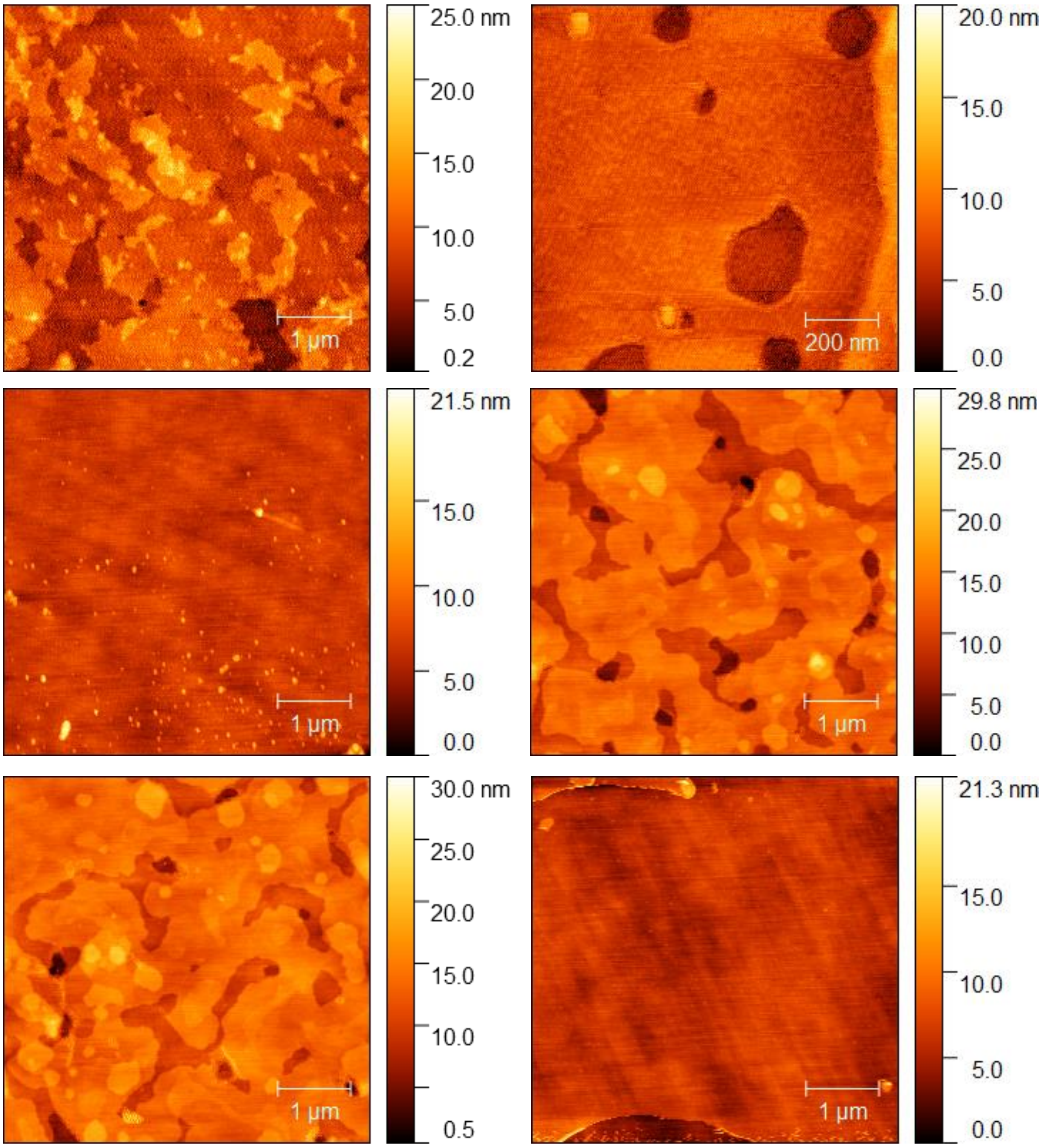

Figure S2: Several representative AFM images of the multi-SLB films coated on silicone oxide surfaces. As can be observed in the figures, we can find different topographies at different areas of the film.

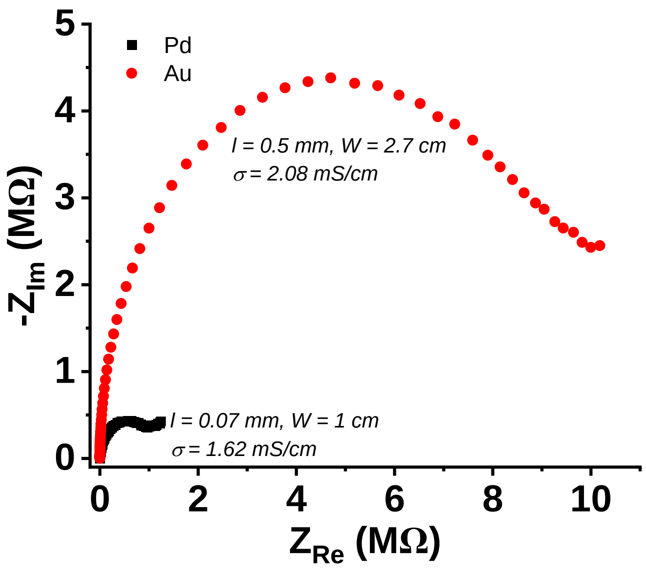

Figure S3: Nyquist plot comparing the conductivities resulting from EIS measurements of DMPG (~20 nm thickness) on Au and Pd electrodes (l and W are the length between the electrodes and width of the sample, respectively, and σ is referring to the conductivity extracted using the equation, $\sigma = l/(R\times A)$ where $A= W\times t$). Note that due to the different geometry of the devices, the magnitude of the impedance that is translated to resistance is different, but taking into account the different geometry, the calculated conductivity (or resistivity) is similar in its magnitude.

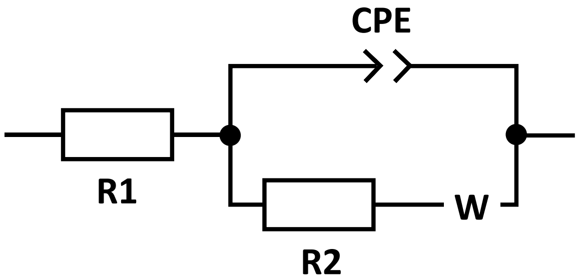

Figure S4: Equivalent circuit used for fitting the Nyquist plots of all the lipids. R1 and R2 represent the contact and bulk resistance, respectively. The constant phase element (CPE) describes the capacitance developed as an electrical double layer next to the electrodes and the Warburg element is the diffusion element.

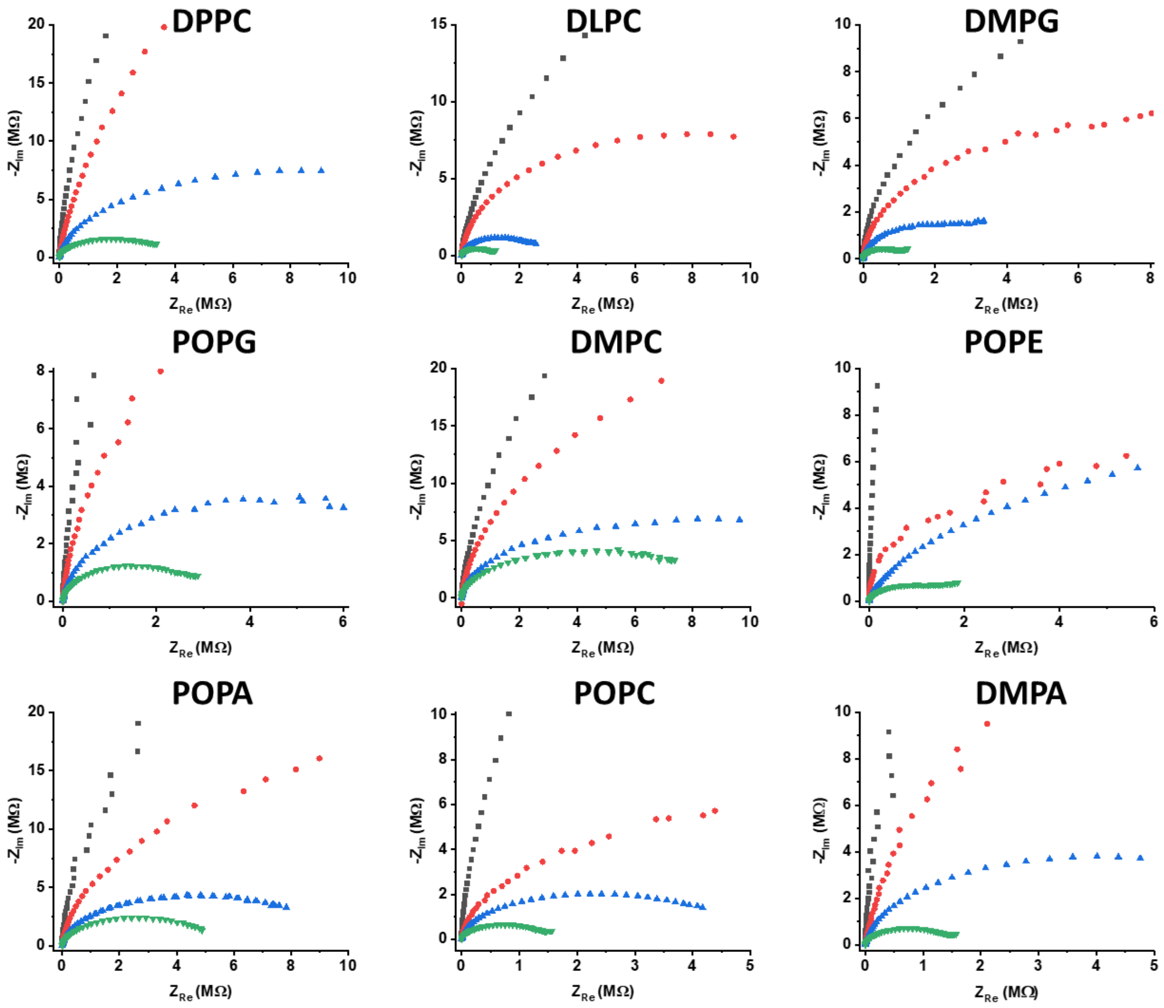

Figure S5: Relative humidity dependent impedance measurements of all the lipids carried out at room temperature. Green: 80% RH, Blue: 70% RH, Red: 60% RH, and Black: 50% RH.

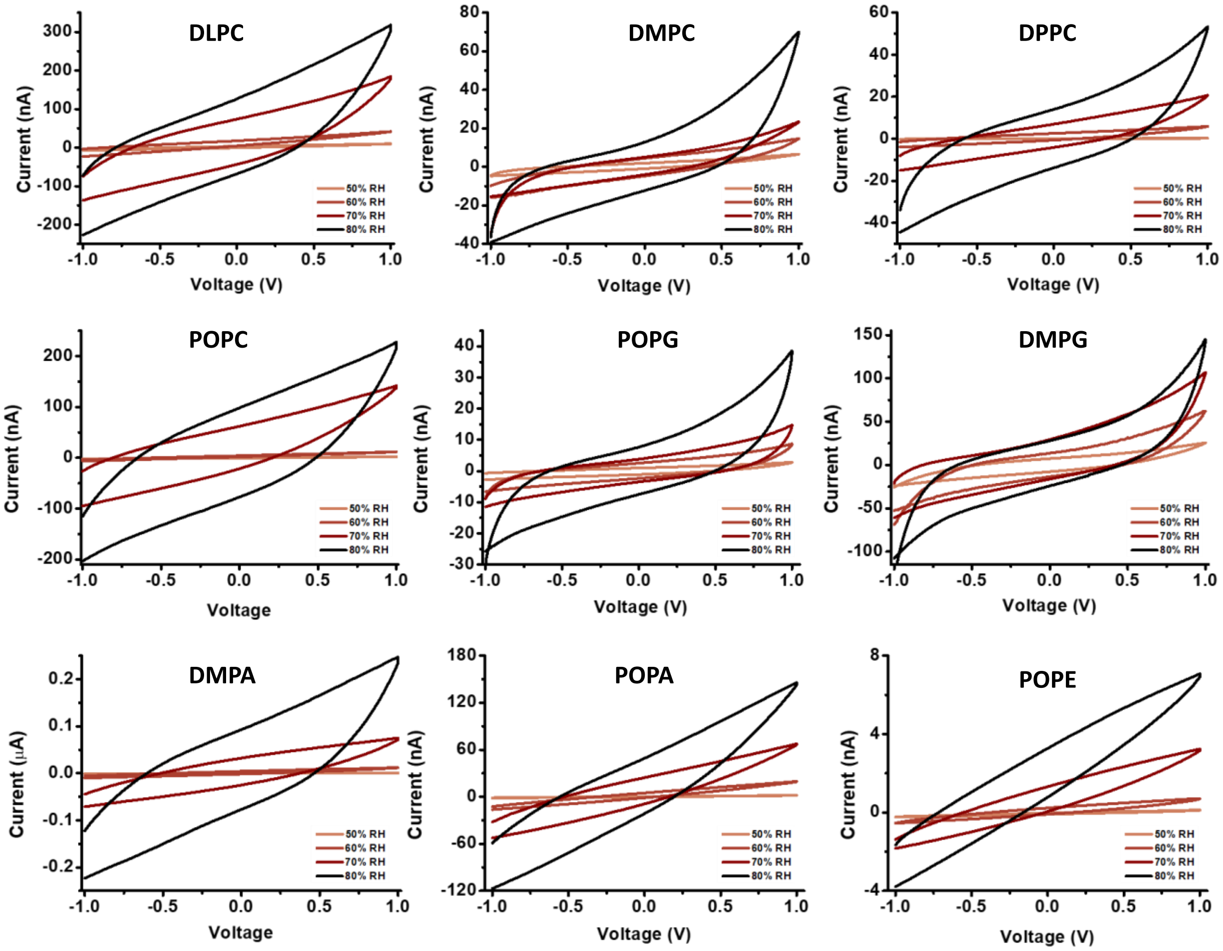

Figure S6: I-V diagrams of all the lipids at different relative humidity conditions (measured at rt).

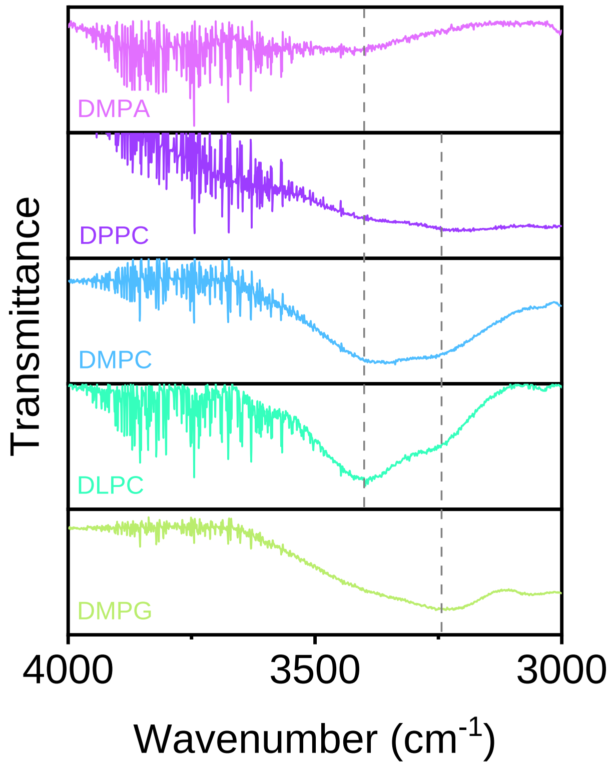

Figure S7: FTIR diagram of saturated lipid membranes showing two important vibrational bands (at 3400 cm^{-1} and 3250 cm^{-1}) between the spectral range of 4000-3000 cm^{-1}.

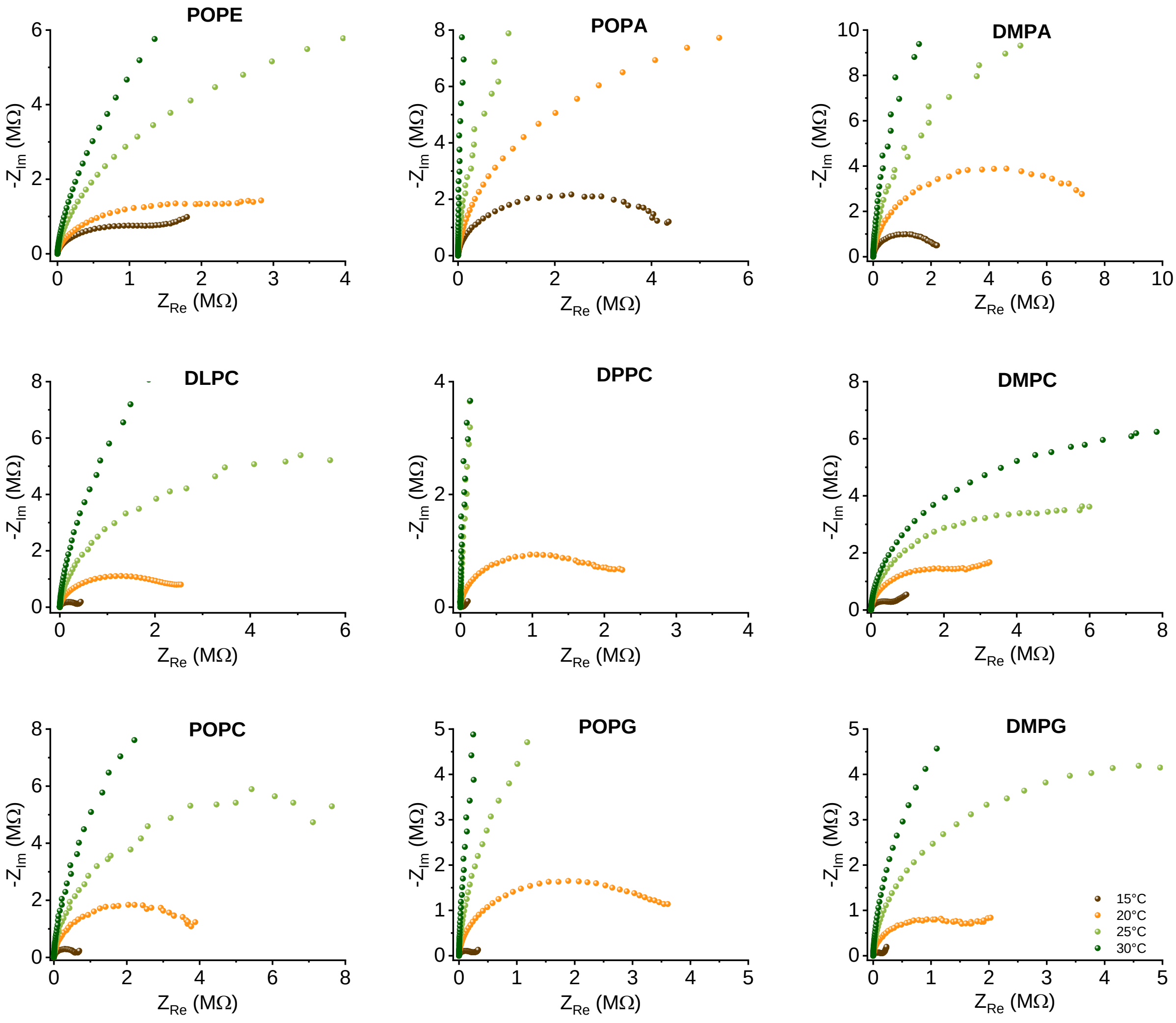

Figure S8: Temperature dependent impedance measurements of all the lipids carried out at 60% RH. Dark green: 15°C, light green: 20°C, orange: 25°C, brown: 30°C

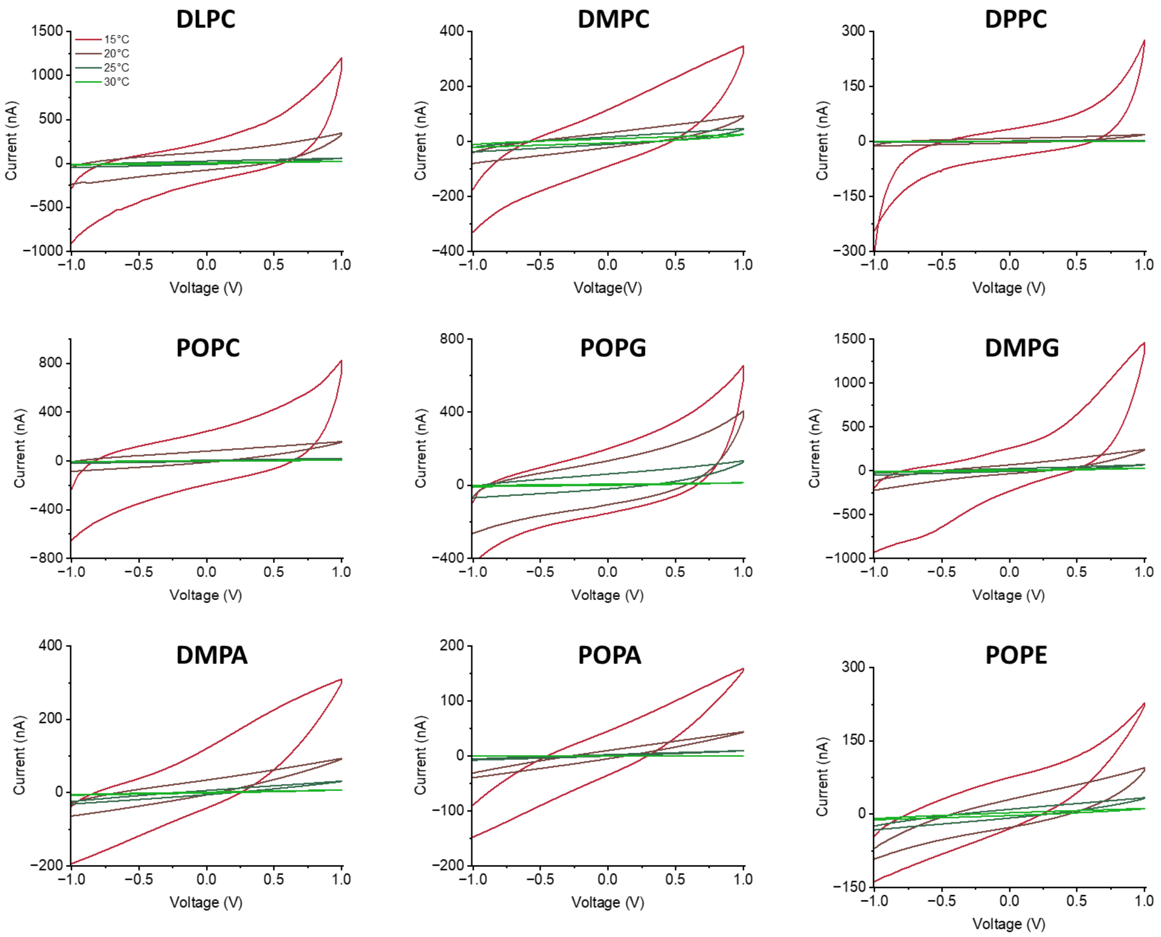

Figure S9: Temperature dependent I-V diagrams of all the lipids at 60% RH.

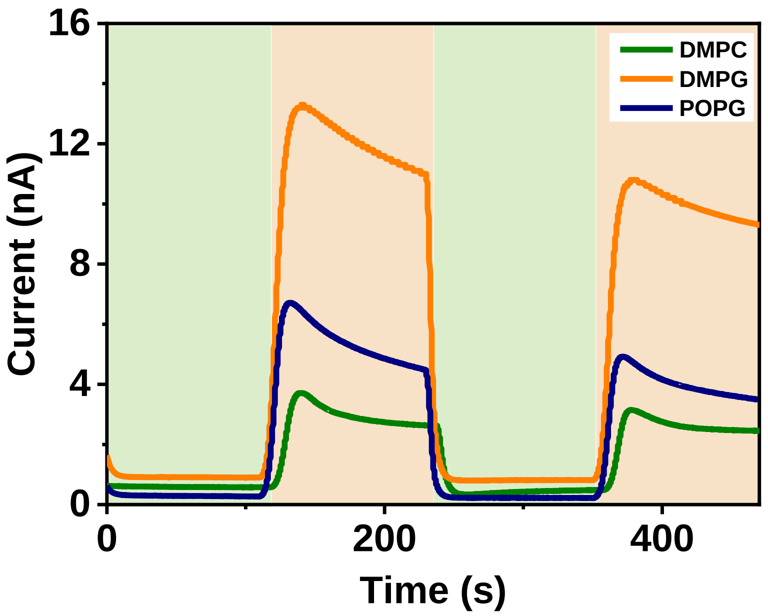

Figure S10: A current-time plot showing the reversibility of currents produced by different SLBs films over consecutive heating and cooling cycles (measured at 0.5 V and 60% RH). Light green represents 30°C and light orange represents 20°C temperature.

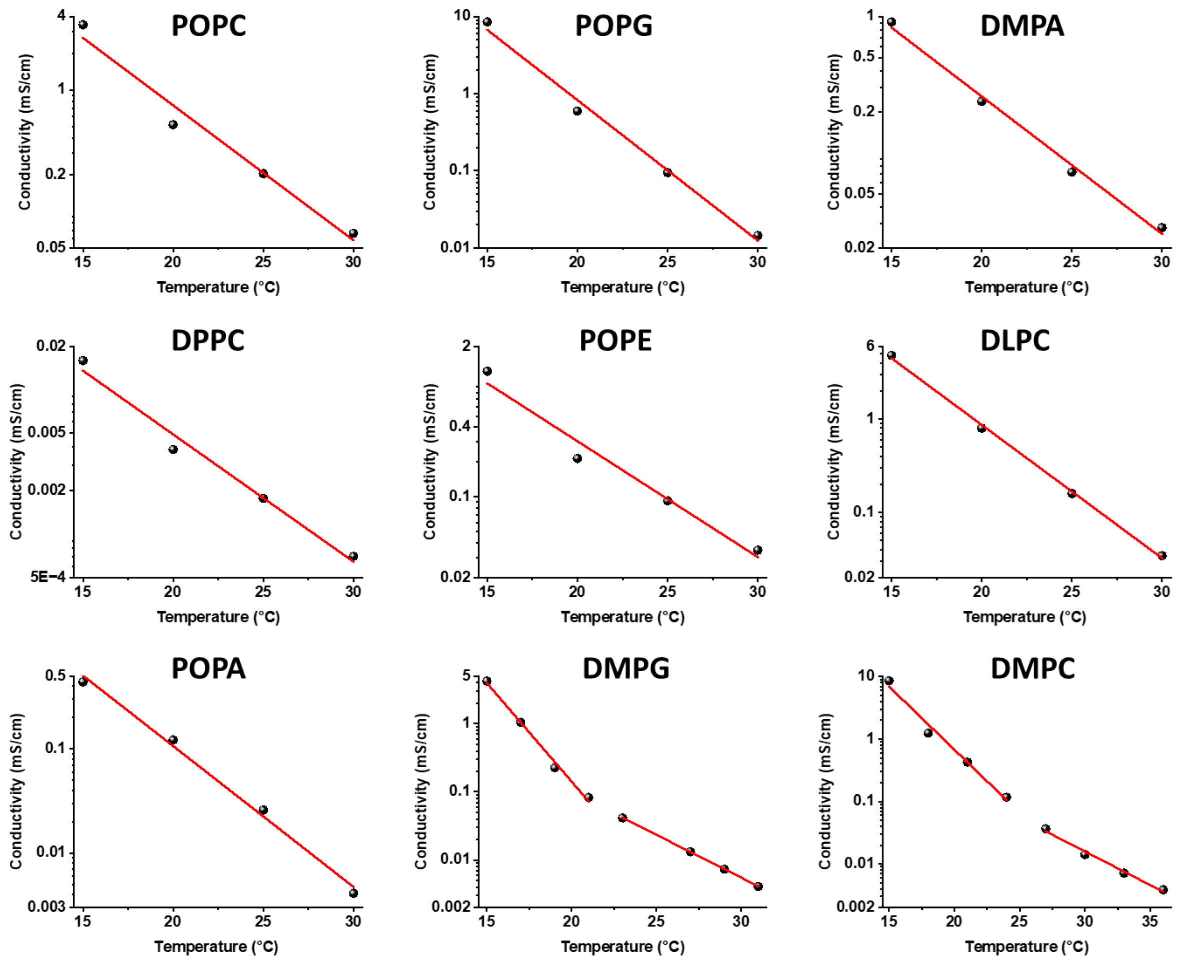

Figure S11: Conductivity extracted from Nyquist plots vs temperature.

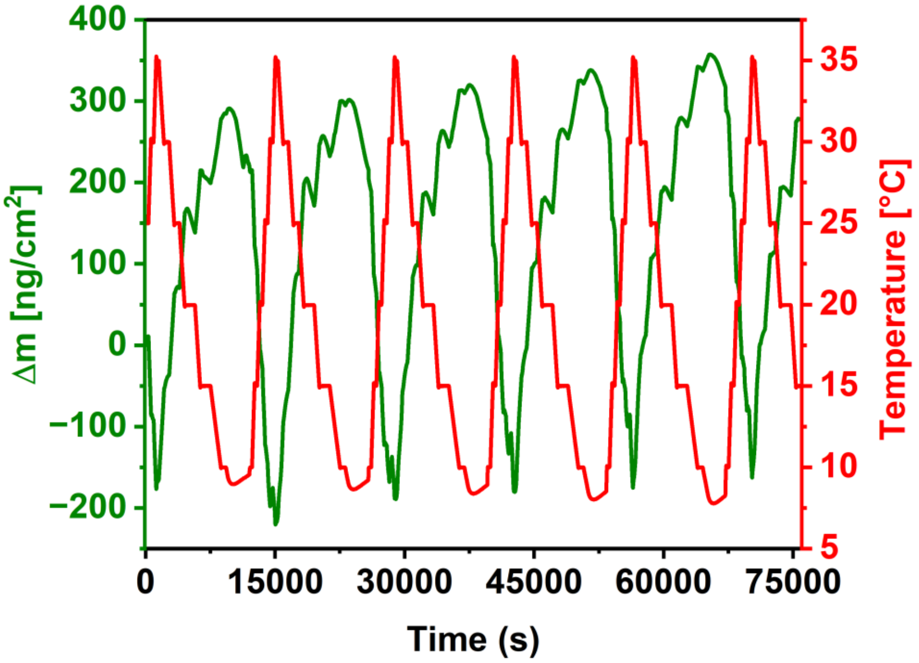

Figure S12: Reversible change in the mass of lipid film, DMPG, over consecutive six heating and cooling cycles at 60% RH.

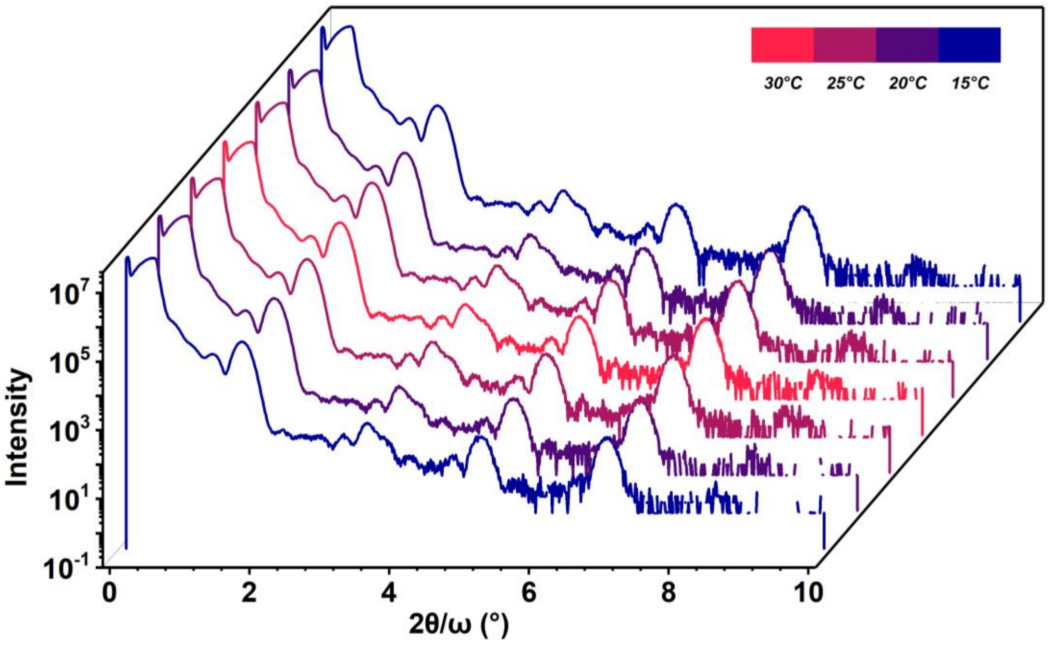

Figure S13: XRR images of DMPG membrane system at different temperatures and @ 60% RH.

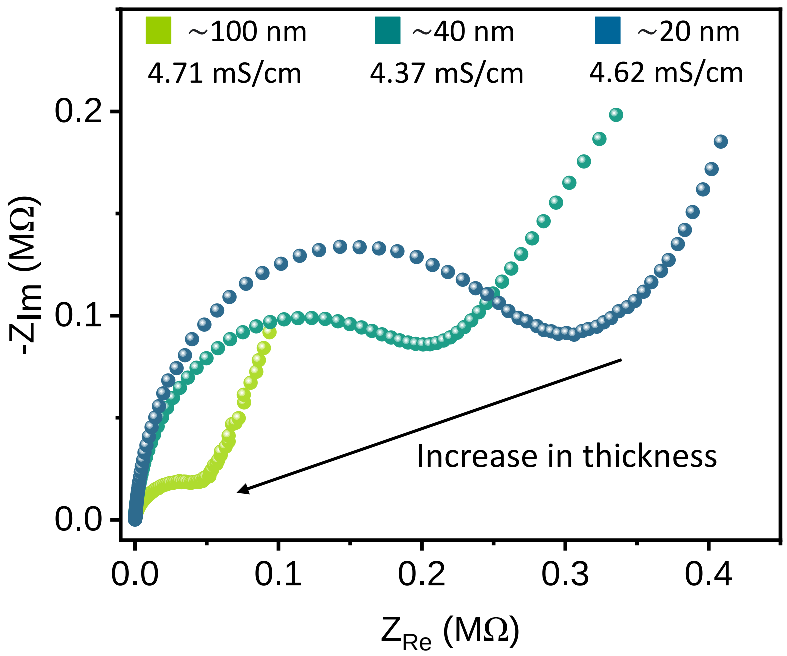

Figure S14: Nyquist plots resulted from EIS experiments of three DLPC SLBs which are different in terms of thickness. Measurements were carried out at 80% RH and rt conditions. The figure also shows the extracted conductivity using the equation, $\sigma = l/(R \times A)$ where $A = W \times t$) and l is the thickness presented in the figure. Note that due to the different thicknesses of the device, the magnitude of the impedance is different, but taking into account the thickness, the calculated conductivity (or resistivity) is similar.
